# Supplementary figures and images for: Coevolutionary Analysis Reveals a Conserved Dual Binding Interface between Extracytoplasmic Function σ Factors and Class I Anti-σ Factors
Source: mSystems. 2020 Aug 4;5(4):e00310-20. doi: 10.1128/mSystems.00310-20 (PMC7406223; doi:10.1128/mSystems.00310-20)

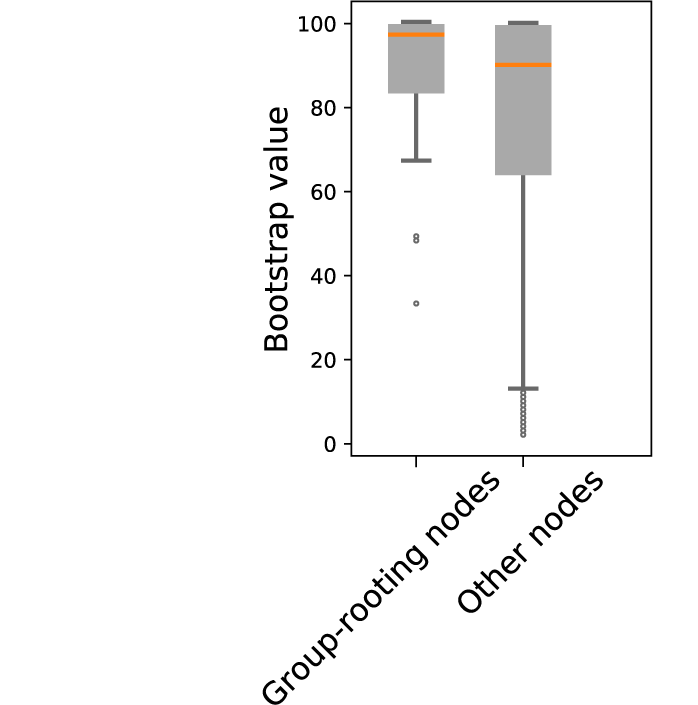

Supplement: FIG S1 [file mSystems.00310-20-sf001.tif]

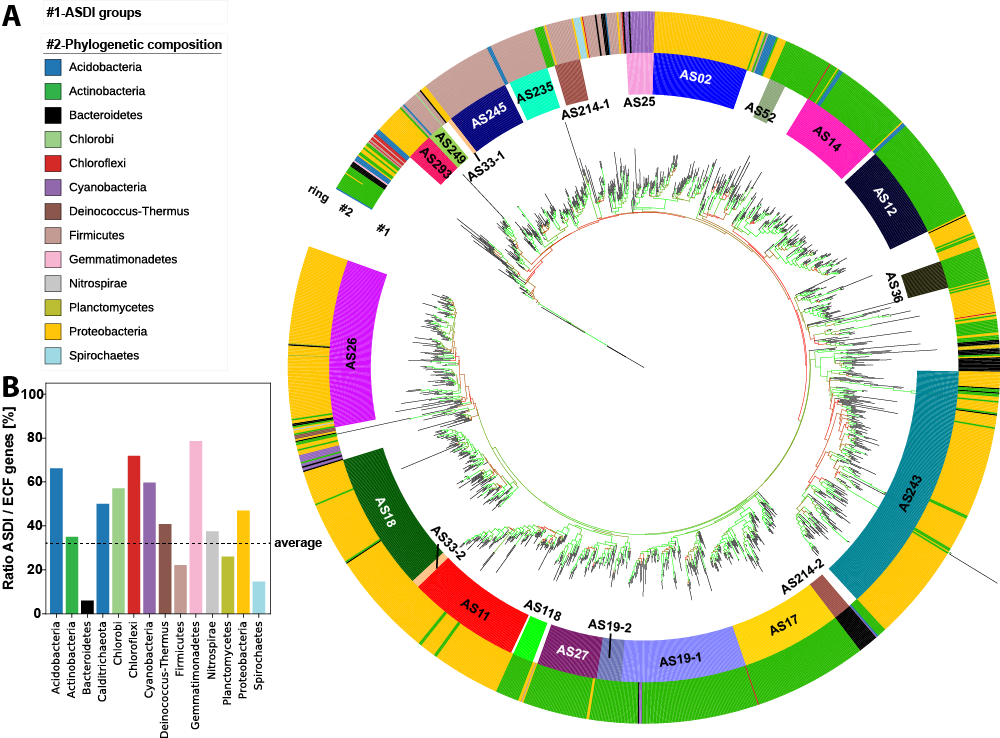

Supplement: FIG S2 [file mSystems.00310-20-sf002.tif]
